# Characterisation of CART-containing neurons and cells in the porcine pancreas, gastro-intestinal tract, adrenal and thyroid glands

**DOI:** 10.1186/1471-2202-8-51

**Published:** 2007-07-11

**Authors:** Nils Wierup, Anna Gunnarsdóttir, Eva Ekblad, Frank Sundler

**Affiliations:** 1 Department of Experimental Medical Science, Lund University, Lund, Sweden; 2 Department of Pediatric Surgery, Lund University Hospital, Lund, Sweden

## Abstract

**Background:**

The peptide CART is widely expressed in central and peripheral neurons, as well as in endocrine cells. Known peripheral sites of expression include the gastrointestinal (GI) tract, the pancreas, and the adrenal glands. In rodent pancreas CART is expressed both in islet endocrine cells and in nerve fibers, some of which innervate the islets. Recent data show that CART is a regulator of islet hormone secretion, and that CART null mutant mice have islet dysfunction. CART also effects GI motility, mainly via central routes. In addition, CART participates in the regulation of the hypothalamus-pituitary-adrenal-axis. We investigated CART expression in porcine pancreas, GI-tract, adrenal glands, and thyroid gland using immunocytochemistry.

**Results:**

CART immunoreactive (IR) nerve cell bodies and fibers were numerous in pancreatic and enteric ganglia. The majority of these were also VIP IR. The finding of intrinsic CART containing neurons indicates that pancreatic and GI CART IR nerve fibers have an intrinsic origin. No CART IR endocrine cells were detected in the pancreas or in the GI tract. The adrenal medulla harboured numerous CART IR endocrine cells, most of which were adrenaline producing. In addition CART IR fibers were frequently seen in the adrenal cortex and capsule. The capsule also contained CART IR nerve cell bodies. The majority of the adrenal CART IR neuronal elements were also VIP IR. CART IR was also seen in a substantial proportion of the C-cells in the thyroid gland. The majority of these cells were also somatostatin IR, and/or 5-HT IR, and/or VIP IR.

**Conclusion:**

CART is a major neuropeptide in intrinsic neurons of the porcine GI-tract and pancreas, a major constituent of adrenaline producing adrenomedullary cells, and a novel peptide of the thyroid C-cells. CART is suggested to be a regulatory peptide in the porcine pancreas, GI-tract, adrenal gland and thyroid.

## Background

The neuropeptide cocaine- and amphetamine-regulated transcript (CART) is highly expressed in the brain [1-7] and exhibits anorexigenic properties [[8,9] for review see [10]]. CART is also found in the peripheral nervous system, including sympathetic preganglionic [11,12], primary sensory [13], enteric [[14-16], for a review see [17]], and pancreatic neurons [18,19], as mostly studied in rodents. In addition, CART is expressed in endocrine cells, e.g. pituitary endocrine cells [3,20], adrenomedullary cells [[3,13,20], for a review see [21]], islet δ-cells [18,22], and antral gastrin cells [15], as studied mostly in rats. During rat development, islet CART expression is not limited to δ-cells, but is evident also in the β-cells, α-cells, and PP-cells [18]. We have recently demonstrated that CART is a regulator of islet hormone secretion and that CART is upregulated in the β-cells of type-2 diabetic rats [23]. We have also shown that CART knock out mice have impaired glucose tolerance and blunted insulin response to glucose, explained by defects at the islet level [[19], for a review see [24]]. Interestingly, humans with a mis-sense mutation in the *cart *gene are obese and are prone to develop type-2 diabetes [25].

CART has been localised to the enteric nervous system (ENS) of several species, including man [for a review see [17]]. In the rat gastrointestinal tract CART is highly expressed in myenteric neurons, and CART immunoreactive (IR) fibres are abundant in the myenteric plexus while they are few in the mucosa [15]. A similar distribution of CART neurons is seen in human GI-tract, although a more sparse innervation of the submucosa and the mucosa is noted [16].

The pig is an important research animal and, except for non-human primates, in many aspects one of the most similar to humans [26,27]. So far, very little is known about the expression and function of CART in the pig. CART containing neurons have been found in the porcine ENS [28]; however, detailed characterisation of these neurons is lacking. Further, although vagal stimulation triggers CART release from the porcine pancreas [28], the porcine pancreatic source of CART is to date not known. Moreover, it is not known whether CART is expressed in porcine adrenal or thyroid glands. The aim of the present study was to perform a detailed mapping of CART in the porcine pancreas, GI-tract, adrenal, and thyroid glands using immunocytochemistry (ICC). In order to further characterise the CART immunoreactive (IR) endocrine cells, nerve fibers and nerve cell bodies, colocalisation of CART with established endocrine or neuronal markers was given special attention.

## Materials and methods

### Animals and tissue processing

Adult domestic pigs (age: 17 months, n = 10) of both genders were used. The animals were killed for other purposes and biopsies were taken from the pancreas, adrenal glands, thyroid gland, stomach (antrum and fundus), small (duodenum and jejunum) and large (caecum) intestine. The specimens were immediately fixed overnight in Stefanini's solution (2% paraformaldehyde and 0.2% picric acid in 0.1 M phosphate buffered saline, pH 7.2), rinsed thoroughly in Tyrode's solution containing 10% sucrose, and frozen on dry ice. Sections (10 μm thickness) were cut and thaw-mounted on slides. The experiments were approved by the animal ethics committee in Malmö and Lund.

### Immunocytochemistry

Antibodies were diluted in phosphate buffered saline (PBS) (pH 7.2) containing 0.25% bovine serum albumin and 0.25% Triton X-100. Sections were incubated with previously characterised primary antibodies overnight at 4°C. The following primary antibodies were used: rabbit anti-CART, code 12/D, dilution 1:1280 (Cocalico Corp., Reamstown, PA) [4,15,18]; rabbit anti-CART, code H-003-62, dilution 1:3000 (Phoenix, Belmont, CA) [29]; mouse monoclonal anti-VIP, code MaVIP, dilution 1:1200 (East Acres Biologicals, Southbridge, MA) [30]; mouse monoclonal anti-tyrosine hydroxylase (TH), code 22941, dilution 1:200 (Incstar, Stillwater, MN) [18]; guinea pig anti-CGRP, code M8513, dilution 1:640 (EuroDiagnostica, Malmö, Sweden) [18]; sheep anti-neuronal nitric oxide syntase (NOS), code AB1529, dilution 1:1600 (Chemicon International Inc., Temecula, CA) [31]; rabbit anti neuropeptide K (NPK), code NPK4, dilution 1:600 (kind gift from Dr E. Theodorsson, The Karolinska Institute, Stockholm, Sweden) [32]; guinea pig anti-phenylethanolamine N-methyl transferase (PNMT), code M8803, dilution 1:1280 (EuroDiagnostica) [33]; rabbit anti-calcitonin, code 7714, dilution 1:640 (EuroDiagnostica) [34]; mouse monoclonal anti-somatostatin, code V 1169, dilution 1:200 (Biomeda, Foster City, CA) [35]; goat anti-serotonin (5-HT), code 20079, dilution 1:1200 (Immunostar, Hudson, WI). The sections were rinsed two times in PBS with Triton X-100 for 2 × 10 min. Thereafter secondary antibodies with specificity for rabbit-, guinea pig-, sheep- or mouse-IgG, and coupled to either fluorescein isothiocyanate (FITC), or Texas-Red (TxR) (Jackson, West Grove, PA), were applied on the sections. Incubation was for 1 h at room temperature. The sections were again rinsed and then mounted in PBS:glycerol, 1:1. The specificity of immunostaining for CART was tested using primary antisera pre-absorbed with excess amount of homologous antigen (100 μg of peptide per ml antiserum in working dilution), or by omission of primary antibodies. Double immunofluorescence was also used, with combinations of primary antibodies (rabbit antibodies, in combination with guinea pig, sheep, or monoclonal antibodies), diluted as described above. The two primary antibodies were incubated simultaneously overnight at 4°C, followed by rinsing in PBS with Triton X-100 for 2 × 10 min. Thereafter the two secondary antibodies were incubated simultaneously for 1 h at room temperature. The following double immunostainings were performed: rabbit CART + anti-rabbit FITC/mouse monoclonal VIP + anti-mouse TxR, rabbit CART + anti-rabbit FITC/guinea pig CGRP + anti-guinea pig TxR, rabbit CART + anti-rabbit FITC/mouse monoclonal TH + anti mouse TxR, rabbit CART + anti-rabbit TxR/guinea pig CGRP + anti-guinea pig FITC, rabbit CART + anti-rabbit TxR/sheep NOS + anti-sheep FITC, rabbit CART + anti-rabbit TxR/mouse monoclonal VIP + anti-mouse FITC, rabbit CART + anti-rabbit TxR/guinea pig PNMT + anti-guinea pig FITC, rabbit CART + anti-rabbit TxR/mouse monoclonal somatostatin + anti-mouse FITC, rabbit CART +anti-rabbit FITC/goat 5-HT + anti-goat TxR. In these studies the controls included tests for inappropriate binding of the secondary antibodies. Double staining for CART/NPK and CART/calcitonin were not possible due to lack of appropriate antibodies, therefore testing of colocalisation of these immunoreactants were performed on consecutive sections. All antibodies, except for the VIP antibody, were raised against synthetic peptides or to peptides from other species than the pig. The CART antibodies used in these studies are directed to 79–102 and 55–102 of rat long CART. Amino acid sequence alignment analysis, using ClustalW 1.83-software, revealed that within these regions the amino acid sequence of pig CART and rat CART are identical. Alignment analysis also showed a high degree of homology (>90%) between species for CGRP, PNMT, NPK, NOS, calcitonin, somatostatin, and TH.

### Image analysis

Immunofluorescence was examined in an epi-fluorescence microscope (Olympus, BX60). By changing filters the location of the different secondary antibodies in double staining was determined. Images were captured with a digital camera (Olympus, DP50). Degree of colocalisation between CART immunoreactivity (IR) and IR for VIP, CGRP, PNMT, somatostatin, or 5-HT was quantified in at least 3 sections of each specimen from 4 pigs. Data are presented as means ± SEM.

## Results

### Immunocytochemistry

#### Pancreas

**Figure 1:**
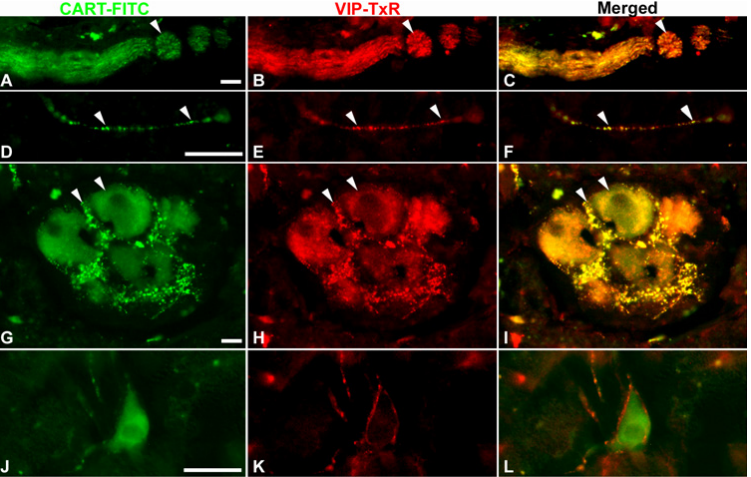
Nerve fibers and ganglia in porcine pancreas double immunostained for CART (A, D, G, J) and VIP (B, E, H, K); merged in C, F, I, L. A–C: Large nerve trunk. D–F: delicate nerve fiber. G–I: Intrapancreatic ganglion. Note high degree of colocalisation of CART and VIP in nerve fibers and nerve cell bodies. J–L: CART IR nerve cell body devoid of VIP, but surrounded by VIP IR fibers. Colocalisation exemplified with arrowheads. Scale bars = 20 μm.

**Figure 2:**
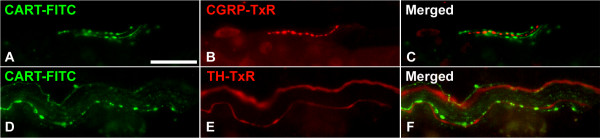
Nerve fibers in porcine pancreas double immunostained for CART (A) and CGRP (B); merged in C, and CART (D) and TH (E); merged in F. CART IR fibers are co-running with CGRP IR and TH IR fibers, but these fibers are distinct from those containing CART. Scale bars = 20 μm.

CART IR was found in numerous nerve fibers, innervating islets, ganglia and exocrine tissue. To characterize these CART containing fibers with respect to additional transmitters we double stained for VIP, CGRP, and TH. This revealed that 99 ± 1% of the CART IR fibers were also VIP IR (Fig 1A–L). Only a minority (<1%) of the CART IR fibers were devoid of VIP (not shown), and VIP IR fibers devoid of CART were also few (Fig 1J–L). CART IR fibers were often co-running with CGRP IR fibers, but no colocalisation could be verified (Fig 2A–C). CART IR fibers clearly differed from TH IR fibers, but the two types of fibers were often co-running (Fig 2D–F). A great proportion of the intrapancreatic ganglia contained nerve cell bodies that were CART IR. Double staining for CART and VIP revealed a high degree of colocalization in such cell bodies (Fig 1G–I). However, CART IR ganglionic nerve cell bodies devoid of VIP were regularly detected (Fig 1J–L). A few CART IR nerve cell bodies were also NOS IR (data not shown). No CART IR islet cells were detected. Preabsorption of the CART antibodies with CART 55–102 peptide blocked all neuronal CART staining (data not shown).

#### GI-tract

**Figure 3:**
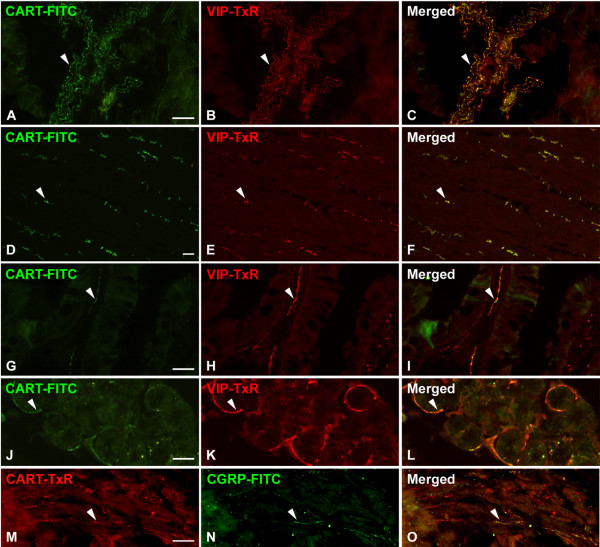
Nerve fibers in porcine GI tract immunostained for CART (A, D, G, J, M) and VIP (B, E, H, K) or CGRP (N); merged in C, F, I, L, O. A–F: jejunal muscularis externa. G–I: duodenal villi. J–L: duodenal glands of Brunner. M–O: muscularis mucosae of the antrum. Note high degree of colocalisation of CART and VIP in all GI segments, except for the muscularis mucosae, where CART is colocalised with CGRP. Colocalisation exemplified with arrowheads. Scale bars = 50 μm.

**Figure 4:**
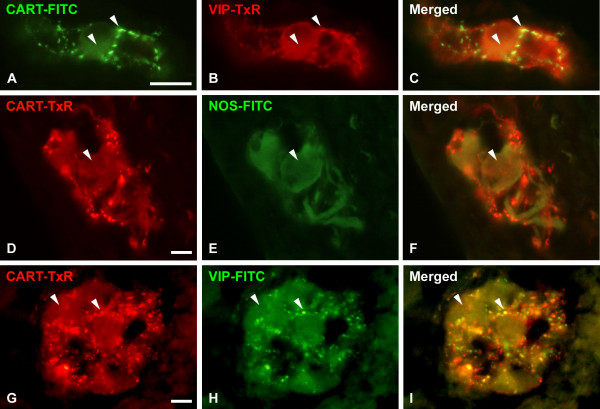
Ganglia in the porcine GI-tract. A–C: jejunal submucous ganglion immunostained for CART (A), and VIP (B), merged in C. D–F: Myenteric ganglion in antrum of the stomach immunostained for CART (D) and NOS (E), merged in F. G–I: Myenteric ganglion of the ileum immunostained for CART (G) and VIP (H), merged in I. Note that CART IR is colocalised with both NOS IR and VIP IR in nerve cell bodies and fibres innervating the nerve cell bodies. Colocalisation exemplified with arrowheads. Scale bar = 25 μm.

Stainings for CART were performed on sections from fundus and antrum of the stomach, duodenum and jejunum of the small intestine and caecum of the large intestine. In all GI-sections studied, CART IR nerve fibers were abundant in the external muscular layers, often in large nerve trunks (Fig 3A and 3D). CART IR fibers were also frequently seen in the submucosa of all specimens, and regularly found to innervate submucous ganglia (Fig 4A). In addition, delicate CART IR fibers were seen in the mucosa of all regions; the density of such fibers was higher in the distal parts of the GI-tract. Thus in the gastric fundus region they were few, while in the duodenum and jejunum CART IR fibers were commonly seen in the core of villi (Fig 3G). CART IR fibers also innervated intra-mucosal nerve cell bodies, which were identified by virtue of their tachykinin (NPK) IR [36] (data not shown). In duodenum CART IR fibers were also abundant in the glands of Brunner (Fig 3J). Double staining for CART and VIP revealed that 95 ± 3% of the CART IR nerve fibers contained also VIP (Fig 3A–L). However, in all segments a small portion of the CART IR fibers in the muscularis mucosae were devoid of VIP. Double staining for CART and CGRP revealed coexistence in a population of fibers in the muscularis mucosae; 60 ± 1% of the CART IR fibers in the muscularis mucosae were also CGRP IR (Fig 3M–O). The majority of these CART IR/CGRP IR fibers were devoid of VIP (not shown).

Further, CART IR nerve cell bodies were found in submucous and myenteric ganglia of all GI sections. In both types of ganglia the majority (99 ± 1%) of these cells were also VIP IR (Fig 4). In addition, myenteric nerve cell bodies harbouring both CART IR and NOS IR were frequently seen (Fig 4D–F). No CART IR endocrine cells were detected in the GI-tract.

#### Adrenal glands

**Figure 5:**
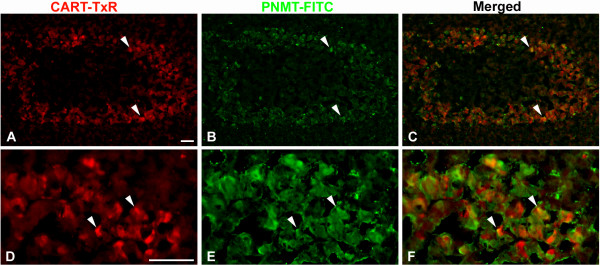
Porcine adrenal gland immunostained for CART (A, D) and PNMT (B, E); merged in C and F. CART is present in a major subpopulation of the PNMT IR, i.e. adrenaline producing, medullary endocrine cells. Colocalisation exemplified with arrowheads. Scale bars = 100 μm.

**Figure 6:**
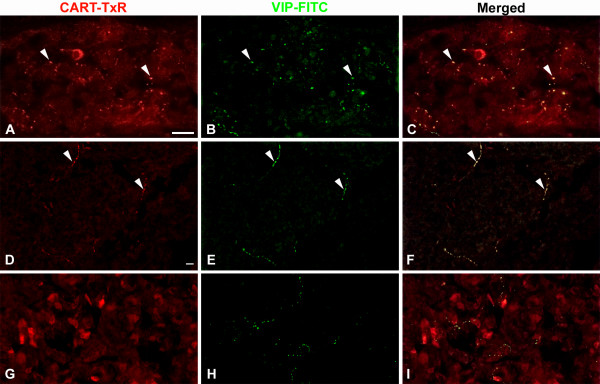
Porcine adrenal gland immunostained for CART (A, D, G) and VIP (B, E, H); merged in C, F, and I. A–C: CART is colocalised with VIP in fibers within a ganglion in the capsule. Note also CART containing nerve cell bodies devoid of VIP. D–F: CART is colocalised with VIP in fibers in the cortex. G–I: In the medulla most VIP IR fibers are devoid of CART. Colocalisation exemplified with arrowheads. Scale bars = 20 μm.

Numerous CART IR cells were found in the adrenal medulla. These cells were mainly located in the peripheral parts of the medulla, and double immunostaining for PNMT, a marker for adrenaline producing cells, revealed that the majority (98 ± 1%) of the CART IR cells were also PNMT IR (Fig 5A–C). In addition, CART IR fibers were frequently seen in the adrenal cortex and in the capsule; the latter also harboured small ganglia with CART IR nerve cell bodies (Fig 6). Double staining for CART and VIP revealed that 90 ± 2% of the CART IR fibers running in the cortex and in the capsule were also VIP IR (Fig 6A–F). Regularly CART IR nerve cell bodies devoid of VIP were seen in nerve cell bodies in the capsule (Fig 6A–C). Only few CART IR fibers were seen in the medulla, while VIP IR fibers were numerous (Fig 6G–I).

#### Thyroid gland

**Figure 7:**
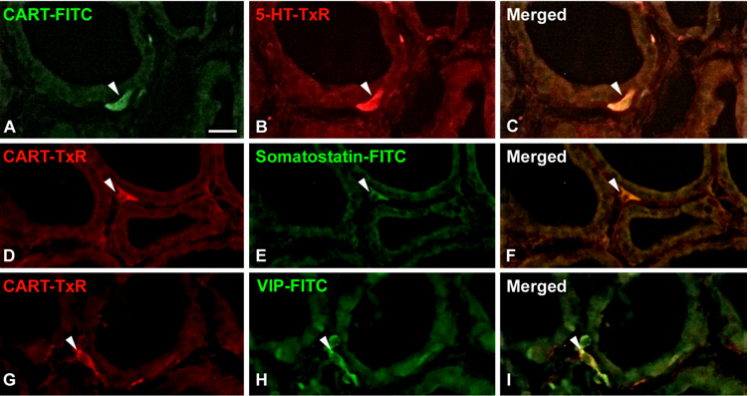
Porcine thyroid gland immunostained for CART (A, D, G), 5-HT (B), somatostatin (E), and VIP (H); merged in C, F, I. CART, which is expressed in the C-cells, is to a varying degree colocalised with 5-HT, somatostatin, and VIP. Colocalisation exemplified with arrowheads. Scale bar = 20 μm.

CART IR cells were also found within the thyroid gland (Fig 7). These cells were located parafollicularly and were identical to C-cells, as revealed by staining for calcitonin on consecutive sections. Double stainings for CART/somatostatin, CART/5-HT, and CART/VIP revealed that 82 ± 4% of the CART IR cells were also somatostatin IR, 65 ± 8% of the CART IR cells were also 5-HT IR, and 60 ± 10% of the CART IR cells were also VIP IR. Only very few CART IR nerve fibers were detected in the gland, some of them were also VIP IR or 5-HT IR (not shown).

## Discussion

CART expression has previously been found in neurons and endocrine cells in the pancreas and in the GI-tract of rodents [15,18,19,37] and humans [16](Wierup et al Ms in preparation). CART has also been localised to nerves and endocrine cells in the adrenal medulla of rodents [3,13,20]. Here we demonstrate that CART is abundantly expressed in neurons in the porcine pancreas, adrenal gland, and GI-tract, as well as in adrenomedullary endocrine cells. Further, we show for the first time that CART is produced in C-cells in the porcine thyroid.

### Pancreas

CART IR neurons were characterized by the use of established markers for the various nerve types in the pancreas. The vast majority of the CART IR fibers and cell bodies were also VIP IR. This observation is in analogy with our previous findings in rat and mouse pancreas, where CART is present in a prominent proportion of the VIP-containing neurons [18,19]. The presence of CART/VIP-containing nerve cell bodies in local pancreatic ganglia indicates an intrinsic origin of at least a portion of the CART IR fibers. The present finding of colocalisation of CART and NOS in porcine pancreatic nerve cell bodies is in line with previous observations on colocalisation of VIP and NOS in pancreatic neurons of several species [38]. We could not detect any colocalisation of CART and CGRP in pancreatic nerve fibers. This differs from previous findings in rat and mouse pancreas where CART is found in the majority of the extrinsic, CGRP-containing, sensory neurons [18,19]. Similarly to pancreatic CART IR fibers in the rat [18], the fibers in porcine pancreas were distinct from TH-containing, adrenergic fibers. Taken together, our data suggest that in the porcine pancreas CART IR fibers emanate at least in part from local intrapancreatic ganglia.

In contrast to rats, which have CART IR δ-cells [18], no CART IR islet cells were detected in pig. This is, however, similar to adult mice, which have a rich CART innervation, but virtually lack CART IR islets cells [19].

The presence of CART in intrinsic VIP containing neurons suggests that CART is involved in the control of islet function in the pig, since these neurons are known to exert stimulatory actions on insulin secretion [for references see [39]]. We recently showed that CART 55–102 is a regulator of islet hormone secretion [23]. In addition, CART null mutant mice displayed islet dysfunction together with impaired glucose tolerance and blunted glucose stimulated insulin secretion [19]. On the other hand, Tornoe et al [28] were unable to detect any effect of CART 42–89 on insulin or glucagon secretion from perfused porcine pancreas. Further studies are needed to elucidate a possible role for neuronal CART in the regulation of islet function in the pig. Peripherally administered CART has been reported to stimulate pancreatic exocrine secretion in the rat [40]. Interestingly, the effect was abolished after vagotomy and diminished after treatment with atropine. Thus, one function of CART in the intrapancreatic neurons could be to regulate exocrine secretion. Since we found that CART is localised to VIP containing neurons, and since VIP is known to exert stimulatory effects on local blood flow [41] and on exocrine secretion [42], it is not inconceivable that CART may modulate these VIP-induced effects on the exocrine pancreas. In addition, CART in pancreatic neurons may be neuroprotective and/or neurotrophic in situations of stress or injury, since such actions of CART have been observed in certain central and enteric neurons [17,43-45].

### GI tract

CART IR neuronal elements were abundant in all layers of the wall of the intestinal segments studied, with a similar distribution pattern as in the rat [15] and guinea-pig [37], and in agreement with previous preliminary observations in the pig [28]. The distribution of CART in the porcine GI tract differed somewhat from that in the human GI tract where CART IR is only rarely detected in the submucosa or mucosa [16]. The vast majority of the CART IR fibers and nerve cell bodies were also VIP IR. This is similar to our and others observations in the rat [15] and guinea pig [37] and human [16] GI tract. Together these data suggest that the majority of the CART IR fibers have an intrinsic origin, since both VIP IR and CART IR nerve cell bodies are present in local enteric ganglia [for references see [17]]. Interestingly, a subpopulation of the CART IR fibers in the muscularis mucosae was CGRP IR. This is different from the rat, where no such colocalisation could be demonstrated [15]. CGRP has been shown to inhibit spontaneous motor activity of the guinea pig mucularis mucosae [46]. A role for CART in these fibers as a regulator of motor activity of the muscularis mucosae needs further investigation. We were unable to detect any CART containing endocrine cells in the GI-tract. This is also different from rats, which harbour CART in a great proportion of the G-cells in the gastric antrum [15], but similar to the mouse, which lacks CART in the G-cells (own unpublished observations).

The location of CART to VIP containing neurons suggests that CART is involved in GI-motor functions since VIP containing neurons are known to play roles in motor control [27]. Okumura et al [47] reported that centrally administered CART peptide inhibits gastric emptying and gastric acid secretion via corticotropin-releasing factor in rats. Further, Tebbe et al [48] demonstrated that centrally injected CART peptide reduced colonic motility via cholinergic pathways in rats; although these effects may be mediated via central effects and hypothalamic neuropeptides as mediators, a role for endogenous CART in these effects needs further attention. We demonstrated that CART 55–102 provokes inhibition on NO-mediated relaxation in the colon in vitro [15]. Recent findings by Jimenez-Feltström et al [49] suggest that NO action in the rat pancreatic islets can be inhibited by GLP-1 and GIP via activation cAMP/PKA dependent pathway. We recently demonstrated that also CART can activate the cAMP/PKA dependent pathway in islet β-cells [23]. It is not inconceivable, therefore, that the inhibitory effect of CART on effects exerted by NO is mediated via increased cAMP also in the gut. Interestingly, CART has been shown to promote survival of rodent enteric neurons in vitro [17]. Thus, CART in porcine ENS may promote survival and protect GI-neurons in situations of neuronal stress or injury. CART resided also in VIP containing fibers innervating the Brunner glands and the gut mucosa. Since VIP is a well established gut secretagogue [50], a role for CART in modulation of VIP mediated secretory functions cannot be excluded.

### Adrenal glands

Our present finding of CART expression in the porcine adrenal medulla is in line with previous reports in rodents [3,13,20] and suggests a role for CART as a signalling molecule in the sympatho-adrenal axis also in the pig. Further, the localisation of the CART containing cells to the more peripheral parts of the medulla, where adrenaline producing cells predominate [51], is similar to the CART mRNA expression pattern reported by Couceyro et al in the rat [20]. The presence of CART IR fibers in the adrenal cortex is in line with the reported effects of CART on glucocorticoid secretion [52]. CART has been shown to be an important player in the stress response, as studied mainly in rodents [52]. Thus, our data of CART in the porcine adrenal gland suggests that CART may be involved in the stress response also in the pig.

### Thyroid gland

A novel finding in the present report is the existence of CART IR in C-cells in the porcine thyroid. This finding gain further support from similar observations in guinea pig (own unpublished observations). This raises the possibility that CART may play roles in calcium homeostasis by modulating the response of calcitonin, a major regulator of calcium [53]. Interestingly, CART knock out mice displayed lower bone mass and increased number of osteoclasts [54]. Further, increased plasma levels of CART in mice and humans are associated with higher bone mass [55]. Whether CART is expressed in mouse C-cells is not known. It is, however, tempting to speculate that CART modulates calcitonin secretion or action, and that the observed CART knock out phenotype is explained by lack of CART action on calcitonin.

In conclusion, CART is highly expressed in the porcine pancreas, GI-tract, adrenal glands, and thyroid gland. The wide spread expression of CART suggest a role for CART as modulator of neurohormonal functions. The similarities of the pattern of CART expression with that of rodents and humans emphasizes the pig as a potential animal model for future studies aimed at increasing the knowledge about CART distribution and function.

## Authors' contributions

NW designed the study, carried out the majority of the experiments and data collection, made all the figures and drafted the manuscript. AG and EE participated in the design of the study and in revising the manuscript. FS conceived and designed the study, collected data, and revised the manuscript.
